# Biomass production from *Bacillus *sp. RAB9 using several carbon sources

**DOI:** 10.1186/1753-6561-8-S4-P172

**Published:** 2014-10-01

**Authors:** Cívita Sousa, Bruno Klainer, Karine Lima, Gustavo Pinto

**Affiliations:** 1 EMBRAPA Agroindustry Tropical, Fortaleza, CE, Brazil

## Background

The NYD medium (glucose, meat extract, yeast extract and peptone) is used for biomass production of some bacteria species that promote plant species growth [1-3]. The main carbon source present in this medium is the glucose that contributes a percentage of 25% to the medium price. An alternative to obtain a more accessible price for the medium is to replace the glucose by sources of lower cost. Several byproducts and feedstock from food industry and agro industry have been applied on microorganisms growth due your high availability and low cost. Among these, the molasses stands out as culture medium in fermentative processes because of your high contents of sugars, nitrogen and vitamins [4]. This study evaluated the replacement of glucose by comercial sucrose, soy molasses and sugar cane molasses seeking better conditions for biomass production from *Bacillus *sp. RAB9.

## Methods

In this study was used the strain of *Bacillus *sp. RAB9. The assays were performed in 250 ml flasks with 100 ml of medium and incubated at 30°C on an orbital shaker at 150 rpm. The biomass was quantified between 4 and 24 hours. The sugar content in each molasses was determined spectrophotometrically by the DNS method. The biomass was quantified from the absorbance (600 nm) of the medium after centrifugation at 3500 rpm for 15 min using pre defined standard curves. A volume of 0.01 g.L^-1 ^of inoculum, calculated according the standard curves, was inoculated in the fermentation medium. The NYD medium has the following composition (g.L^-1^): glucose 10.0; yeast extract 5.0; meat extract 3.0; peptone 5.0; pH 6.5. Tested: sucrose 10.0; soy molasses 10.0; sugar cane molasses 10.0.

## Results and conclusions

In NYD medium the production biomass was 0.74 g.L^-1 ^in 24 hours of fermentation. The results found showed an increase on lineage growth of 39.2% for sucrose, 95.9% for soy molasses and 310.8% for sugar cane molasses. The replacement by cane molasses contributed to a 76.7% reduction in biomass cost. Selected the sugar cane molasses to replace the glucose in NYD medium was evaluated the concentrations of 10 to 20 g.L^-1 ^for choose the best concentration for replacement and subsequently the best temperature of 30 to 45°C. The increase on cane molasses concentration did not contribute for the gain on biomass. Was observed that the best alternative to substitute glucose is the sugar cane molasses on concentration of 10 g.L^-1 ^at 40°C.
